# Play hard, study hard? The influence of gamification on students’ study engagement

**DOI:** 10.3389/fpsyg.2022.994700

**Published:** 2022-10-10

**Authors:** Jun Chen, Mo Liang

**Affiliations:** ^1^Lifelong Education College, Shanghai Jiao Tong University, Shanghai, China; ^2^USC-SJTU Institute of Cultural and Creative Industry (ICCI), Shanghai Jiao Tong University, Shanghai, China

**Keywords:** gamification, enjoyment, self-efficacy, study engagement, education

## Abstract

In recent years, gamification is widely used in the education. In this article, we build one theoretical model to illustrate how gamification influences students’ study engagement. To examine our hypotheses, we distributed our questionnaire surveys to 187 students from one university of China. Correlational analyzes, regression analyzes and confirmatory factor analyzes were used to test our hypotheses. The results show that gamification influences students’ study engagement through the indirect effects of enjoyment and self-efficacy. Implications and future research directions are discussed.

## Introduction

People play games while traveling, unwinding, or at work to generate delightful experiences. In the modern-day, where social media and digital technology mediate most of what we do, many firms shift that behavior by transforming routine tasks into rich, fun, gaming-like experiences (Wang et al., 2021). This process is called gamification (Robson et al., 2015).

Generally, gamification refers to the functionality of interactive systems that leverage the use and mechanics of game elements to motivate or engage end-users (Seaborn and Fels, 2015). Additionally, gamification refers to technologies aiming to increase intrinsic incentives for various tasks, typically by utilizing game design elements (Hamari and Koivisto, 2015).

Academics, educators, and practitioners have been interested in gamification in various fields, including education, information studies, human−computer interaction, and public health (Seaborn and Fels, 2015). Gamification has thus far been used in a wide range of situations, from consumer behavior and sustainable consumption to exercise (e.g., Fitocracy) and general welfare (e.g., Foursquare). Gamification is a multifaceted socio-technological phenomenon that can offer various advantages, including enjoyment, social benefits *via* communities, and social engagement (Hamari and Koivisto, 2015).

### Gamification in higher education

Gamification’s influence on education and learning might vary based on audience and content qualities. Research shows that students in gamified courses outperformed because they can concentrate on their studies. For example, college students in a gamified cell biology class outperformed their lecture-based counterparts by 40% (Kim et al., 2018).

Starting with unmet demands and using a simple superficial reward-based layer as an introduction to the system is one way to implement gamification. These benefits must be swiftly swapped out with more worthwhile components, such as a story, the ability to pick which pathways to investigate, enjoyable activities, and chances to reflect (Nicholson, 2015). Due to the games’ knowledge to educate and crucial skills such as problem-solving, cooperation, and communication, using educational games as learning aids is a viable strategy. Games have extraordinary driving power; they use a variety of enticing, sometimes without receiving anything in return, only for the fun of it and the chance to win (Dicheva et al., 2015).

The advantages of the digital gaming medium encourage its use in areas other than entertainment (Seaborn and Fels, 2015). In education, gamification has been used to characterize video games in general and digital game-based learning (DGBL) in particular (Seaborn and Fels, 2015). Since education aims to increase students’ motivation and participation, it has high potential for application (Simões et al., 2013). The use of game mechanics to address issues in learning and education is known as gamification, and it refers to various activities and procedures (Kim et al., 2018). Gamification has been a widely used strategy in recent years. However, academic research on gamification in education still needs more effort to fill some important gaps (Rapp et al., 2019).

To examine whether and how gamification in education influences students’ study outcomes (i.e., study engagement), we conduct one questionnaire survey to 187 students from one university of China. Specifically, we propose that students will perceive enjoyment and higher self-efficacy in the gamified courses. Then they will better engage in their study.

## Hypotheses development

### The influence of gamification on students’ enjoyment

Gamification aims to let users enjoy the system (Hamari and Koivisto, 2015). There are two kinds of influences of gamification on students, namely, intrinsic motivation and extrinsic motivation. Traditional incentive mechanisms are generally based on increasing students’ extrinsic motivation; that is, by introducing financial rewards to motivate students (Blohm and Leimeister, 2013). However, when the extrinsic rewards disappear, the students’ learning motivation may decrease unless they find other reasons to continue their study enagement (Nicholson, 2015). Gamification based on information technology improves the motivation and satisfaction of users to continue learn. In addition, gamification technology can continuously record the behavior of individuals in the game, visualize the progress of individuals’ behavior, help to reach achievable personal goals, and provide immediate feedback, enabling users to feel their high performance (Blohm and Leimeister, 2013).

Educational games are developed to achieve real-world learning and educational goals. Gamers can learn while playing the game and reach their goals when they successfully complete tasks in the game. In other words, educational games complete the teaching in the game (Kim et al., 2018).

The concept of gamified education differs from the concept of games. Gamified education describes mature games designed for non-entertainment purposes, where gamified applications use only game elements (Dicheva et al., 2015). Gamification of education aims to create pathways that support learning and problem-solving in games and implement them in real education (Kim et al., 2018). The gamification of educational methods has the advantage of bringing in what matters from the world of video games to increase student engagement without using any particular game. This purpose is to extract game elements and make teaching enjoyable and exciting by adapting game elements and using these elements in the teaching process. Therefore, students learn not by playing a specific game but by learning as a game. Pure learning gradually becomes boring for students, while games are fun and engaging. With the help of gamification, learning becomes progressively more exciting and enriching if students learn as a game (Simões et al., 2013).

Gamification aims to promote students’ psychological outcomes. These psychological outcomes further serve as intermediaries between behavioral effects and gamified value creation. In other words, although the visibility of the system may cause the fluctuation of psychological states and emotions, the designer can regulate the fluctuating behavior to create value (Huotari and Hamari, 2017).

*H1*: Gamification is positively related to students’ enjoyment.

### The influence of gamification on students’ self-efficacy

The definition of gamification focuses on the term “playfulness” (Hamari and Koivisto, 2015). This refers to the technique of turning things into games to make them more engaging or pleasant. Furthermore, gamification’s ultimate aim is frequently tied to utilitarian goals; gamification’s goal is to encourage useful outcomes outside of the gamified system. Gamification services frequently feature a social component as well (Blohm and Leimeister, 2013).

Self-efficacy reflects one’s self-confidence in his/her ability to perform job tasks to achieve a specific outcome (Bandura, 1977). It is a person’s belief in managing their motivations, behaviors, and social environment. In our daily lives, all elements of people’s experiences are impacted by cognitive self-evaluations, including the goals an individual strives to achieve, the level of effort required to achieve those goals and the likelihood of achieving a certain level of behavioral performance.

Gamified education uses game elements such as scoring through games, competing, and achieving learning goals to motivate and engage students (Swacha, 2021). The gamification option is limited to the teacher’s imagination and encourages more efficient and participatory learning. Gamification success comes from competition, both for yourself and for your peers. The learning impact of competition is based on social interaction and students’ desire to see their names at the top of a scoring list or to compare their scores with peer success (Hassan et al., 2021). Extrinsic motivational rewards can be obtained in various ways and can be rewarded through conscious competing goals, allowing intrinsic motivational growth (Banfield and Wilkerson, 2014; Hanus and Fox, 2015).

Thus, the gamification of education will promote a sense of self-efficacy in which students will compete to increase their internal motivation, which in turn will drive them to learn and make academic progress (Banfield and Wilkerson, 2014).

*H2*: Gamification is positively related to students’ self-efficacy.

### The influence of students’ enjoyment on their study engagement

Students’ lack of learning fun is considered the cause of many failures in study engagement (Lumby, 2011). Many teachers have difficulties in class because their students lack motivation and do not actively participate in classroom activities. Because of real-world experiences such as these, learning motivation and study engagement have long interested educators and researchers. Some researchers have found gamification to be effective in inducing psychological and behavioral changes. For example, gamification can cultivate students’ learning motivation and engagement (Kim et al., 2018). Since learning is an internal process, it can only be inferred or reported from data perceived by students. They can only believe that their emotions are related to learning because they think they enjoy it (Lumby, 2011). It can be seen that gamification can be used for effective teaching because it can promote learner participation (Kim et al., 2018).

The interaction between age and time spent using the service further suggests that the younger the user is, the stronger the novelty effect their sense of playfulness. Younger people, while more susceptible to fun interactions, can also get bored faster than maturer users. This finding may mean that young users may be more active in enjoying and accepting gamification education (Koivisto and Hamari, 2014).

In fact, there are few opportunities to enjoy school. Loss of self-awareness through concentration is rare, and pervasive passive learning is considered intolerable and out of touch with educational ideals. Often, students are asked to do tasks they do not understand and feel like they are even getting nowhere. There is little possibility of accumulating forms of social or interactional capital through positive relationships and authentication with adults (Lumby, 2011). A high level of emotional tension is a distinct feature of today’s educational environment. Research suggests that the joy of learning and pride are intrinsically linked to students’ motivation, learning process, school performance, self-identity development, and school well-being. In the context of hierarchical enjoyment, an experiential model, learning enjoyment is assimilated into a third level, which indicates a lower level of school enjoyment of experience (Manasia, 2015). Therefore, it seems plausible that exhausted students underperform because they feel fatigued, irritable, frustrated, or cynical. Of course, students also have positive feelings and attitudes about their learning. For example, they are also proud and motivated by their success and achievement of important goals (Salanova et al., 2010). Their participation is directly related to performance, which offers the possibility of enhancing participation and improving performance by increasing facilitators or reducing barriers (Salanova et al., 2010).

Of course, game proponents should avoid the “spirit of the game,” believing that all games must be conducive to learning. However, the game provides these participants with a straightforward way to assess their understanding or at least makes them realize that they do not understand the principles as much as they thought (Rieber and Noah, 2008). Intrinsically motivated students learn purely out of interest in the course, not just because of the knowledge itself. Therefore, they are more devoted to research. It turns out that positive emotions also produce feelings of well-being (Wang and Li, 2017). Positive emotions induce exploratory behaviors that create learning opportunities and goal achievement. It helps to build persistent resources to widen the thought-action pool. In a practical sense, enhancing students’ positive emotions and private resources can increase their learning engagement (Ouweneel et al., 2011).

Consequently, positive emotions from the gamification education will improve students’ enjoyment of learning, which in turn promotes their study engagement.

*H3*: Students’ enjoyment is positively related to study engagement.

### The influence of students’ self-efficacy on study engagement

Self-efficacy indicates students’ perceptions and beliefs about their ability to complete learning tasks. Self-efficacy beliefs are “people’s judgments about their ability to organize and execute the course of action needed to achieve a specified type of performance” (Bandura, 1977). That is, the student’s confidence that they can accomplish a learning task.

Study engagement refers to the positive and fulfilling experience associated with learning, including energy, focus, and absorption in education (Zhang et al., 2021). Energy reflects a high quality of mental energy in the learning process. Concentration refers to the ability to identify with learning and sustain a commitment to it. Absorption is the feeling of understanding and describes a student’s concentration on the content and enjoyment of the learning process, unconsciously learning (Siu et al., 2014).

When students believe they can complete a task, they are motivated to engage in it. Self-efficacy plays a vital role in students’ engagement in learning. Students with higher self-efficacy are more likely to be engaged behaviorally, cognitively, and motivationally in the classroom, resulting in higher levels of learning engagement (Linnenbrink and Pintrich, 2003). In other words, when a student has sufficient confidence in their abilities, they are more motivated to complete learning tasks and, in the process, feel more accomplished and enjoy the learning process (Li et al., 2018). Therefore, teachers must focus on improving students’ self-efficacy during lessons (Manasia, 2015). For example, by giving positive feedback when students progress and encouraging them when they encounter setbacks in the learning process. During the epidemic, how to improve classroom participation in online classes is a hot issue for research, and improving students’ self-efficacy is a good entry point.

*H4*: Students’ self-efficacy is positively related to study engagement.

### The mediating role of students’ enjoyment

The use of gamification in teaching can activate students’ thinking, enhance students’ communication skills, and help students change their inherent way of thinking. In addition, students can be intrinsically motivated by gamification and have a positive attitude toward gamification teaching in the application (Yu et al., 2022). Thus students can continuously adjust their learning progress and learning methods according to the game elements of the e-learning system, such as rankings, levels, challenges, and tasks, to improve their academic performance and performance in school. Through practice, it has been found that the test scores of the gamified teaching group are higher than those of the non-gamified teaching group, effectively improving the students’ academic performance. Therefore, students will enhance their internal and external motivation and fully enjoy learning because of their novel learning methods, diversified teaching concepts, competitive learning, and collaborative attitudes. In addition, there is a significant correlation between game scores and end-of-semester test scores, so gamification makes students more competitive (Lera et al., 2022). Students will enjoy learning more when they are full of learning competitive advantages.

*H5*: Students’ enjoyment mediates the relationships between gamification and study engagement.

### The mediating role of students’ self-efficacy

Self-efficacy will make students have strong self-confidence, always have confidence in completing tasks and are willing to make efforts to complete them successfully. In addition, the students’ confidence also qualitatively improves with the successful completion of the task (Chiang et al., 2022). Similarly, students with low self-efficacy will immerse themselves in the loss of motivation after facing setbacks and difficulties. The result is that they face failure, and their self-confidence is further reduced. In addition, there is a significant correlation between self-efficacy and learning factors such as academic performance and ability improvement. The strength of self-efficacy will affect students’ academic performance. Students who improve their self-efficacy will be more likely to obtain better professional results and have the ability to self-regulate. Moreover, the strength of self-efficacy affects the level of effort students make to determine behavior. People with high self-efficacy make a tremendous effort to make their own decisions. University students with high self-efficacy have correspondingly higher expectations for learning, and they believe that through their unremitting efforts, they will be able to achieve learning goals that are consistent with their ability levels.

*H6*: Students’ self-efficacy mediates the relationships between gamification and study engagement.

**Figure 1:**
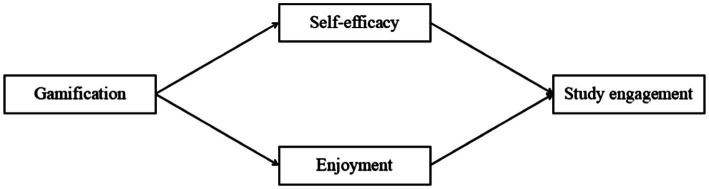
The theoretical model in this study.

Figure 1 shows the theoretical model in this study.

## Materials and methods

### Sample and procedures

To examine our hypotheses, we collected data from one university in China. We distributed our questionnaire surveys to undergraduate students who were enrolling in the Marketing course in 2022. We assured that their participation was voluntarily and that their responses would by used only for research purposes. We distributed our questionnaire surveys to 240 students and received 187 valid responses. The response rate was 78%. The descriptive statistics of the sample are as follows: In terms of gender, 43% are male and 57% are female; in terms of age, 79% are 18–20 years old, 21% are 21–23 years old, and 1% are 23 and older. In terms of household income, below 5,000 yuan accounts for 2%, 5,001–10,000 yuan accounts for 41%, 10,001–20,000 yuan accounts for 48%, 20,001–30,000 yuan accounts for 8%, and 30,000 yuan or more accounts for 2%.

### Measurements

#### Gamification

Gamification was measured using the 6-item scale adapted from Liu et al. (2019). It includes items such as “I feel there was perceivable creative ingenuity in the design of this activity.” Cronbach’s alpha for this scale was 0.87.

#### Self-efficacy

Self-efficacy was measured using the 6-item scale adapted from Speier and Frese (1997). It includes items such as “When I am confronted with a new task, I am often afraid of not being able to handle it (*R*)”; “I judge my abilities to be high”; “If I want to achieve something, I can overcome setbacks without giving up my goal.” Cronbach’s alpha was 0.84.

#### Enjoyment

Enjoyment was measured using the 9-item scale from Botes et al., (2021). It includes items such as “I enjoy it.” Cronbach’s alpha was 0.80.

#### Study engagement

Study engagement was measured using the 9-item scale adapted from Schaufeli et al. (2006). It includes items such as “When I study, I feel like I am bursting with energy.” Cronbach’s alpha was 0.74.

#### Control variables

According to the existing research, we selected several control variables in this study. Participants’ sex, age, and income level are controlled in the following analysis.

## Results

### Confirmatory factor analysis

To examine the discriminate validity of our measurements, we used AMOS 25.0 to conduct confirmatory factor analysis. Table 1 displays the results. The proposed four-factor baseline model provides the best fit index (*χ*^2^/*df* = 1.66 (<3), RMSEA = 0.06 (<0.08), IFI = 0.96 (>0.9), CFI = 0.95 (>0.9), TLI = 0.96 (>0.9)) compared with other alternative models. Therefore, the four constructs can be distinguished well.

**Table 1:** Confirmatory factor analysis results.

Model	Included constructs	*χ* ^2^	*df*	χ^2^/*df*	RMSEA	IFI	TLI	CFI
Single factor	EJ + EN + GA + SE	790.749	104	7.603	0.188	0.531	0.453	0.526
Two-factor	EJ + EN; GA + SE	604.766	103	5.872	0.162	0.658	0.596	0.654
Three-factor	GA; SE; EJ + EN	396.695	101	3.928	0.125	0.798	0.757	0.796
Four-factor	EJ; EN; GA; SE	162.530	98	1.658	0.059	0.956	0.945	0.955

GA, gamification; EJ, enjoyment; SE, self-efficacy; EN, engagement.

### Descriptive statistics

Table 2 presents the descriptive statistics and correlations between the main variables. As shown in the table, gamification was positively correlated with students’ engagement (*r* = 0.26, *p* < 0.01). In addition, gamification was positively correlated with enjoyment (*r* = 0.36, *p* < 0.01), gamification was positively correlated with self-efficacy (*r* = 0.45, *p* < 0.01), enjoyment was positively correlated with study engagement (*r* = 0.40, *p* < 0.01), and self-efficacy was positively correlated with study engagement (*r* = 0.41, *p* < 0.01). There was a significant correlation between enjoyment and self-efficacy (*r* = 0.41, *p* < 0.01).

**Table 2:** Descriptive and correlation analysis of study variables.

	Mean	SD	1	2	3	4	5	6	7
1. Sex	1.57	0.50	–						
2. Income	2.66	0.73	−0.04	–					
3. Age	2.22	0.43	−0.04	0.33^**^	–				
4. Gamification	3.35	1.41	−0.12	0.05	0.03	–			
5. Enjoyment	3.60	0.10	−0.15^*^	−0.01	−0.09	0.36^**^	–		
6. Self-efficacy	4.35	1.29	−0.04	0.03	−0.07	0.45^**^	0.41^**^	–	
7. Study engagement	4.29	0.89	−0.18^*^	0.10	−0.04	0.26^**^	0.40^**^	0.41^**^	–

*N* = 187.

* *p* < 0.05;

** *p* < 0.01.

### Direct effect analysis

As shown in Table 3, we performed a series of multiple regression analyzes (Baron and Kenny, 1986; Hox, 2010) to test our hypotheses. We first examine the direct effects and then the mediating effect. Model 1 examines the effect of control variables on study engagement. Model 2 adds gamification to examine its effect on study engagement. To further test the mediation effect, Model 4 adds enjoyment and gamification to test their effects on study engagement, and Model 8 adds self-efficacy and gamification to test their effects on study engagement.

**Table 3:** Mediating effect analysis.

	Study engagement							
	Model 1	Model 2	Model 3	Model 4	Model 5	Model 6	Model 7	Model 8
**Control variables**								
Sex	−0.18^*^	−0.15^*^	−0.12	−0.11	−0.18^*^	−0.15^*^	−0.16^*^	−0.16^*^
Income	0.12	0.11	0.11	0.11	0.12	0.11	0.09	0.09
Age	−0.08	−0.09	−0.05	−0.05	−0.08	−0.09	−0.04	−0.05
**Independent variable**								
Gamification		0.24^**^		0.13		0.24^**^		0.08
**Mediating variables**								
Enjoyment			0.37^***^	0.33^***^				
Self-efficacy							0.40^***^	0.37^***^
*R* ^2^	0.05	0.11	0.18	0.20	0.05	0.11	0.21	0.21
*ΔR* ^2^	0.03	0.09	0.16	0.17	0.03	0.09	0.19	0.19
*F* value	3.00^*^	5.33^***^	10.08^***^	8.82^***^	3.00^*^	5.33^***^	11.86^***^	9.69^***^

*N* = 187.3

* *p* < 0.05;

** *p* < 0.01;

*** *p* < 0.001.

Table 3 presents the regression analysis data of the main effects and the mediating effect, among which Model 1 and Model 2 represent the main effect. The final result shows that after controlling for the three variables of gender, age, and family income, gamification and engagement are positively correlated (*β* = 0.24, *p* < 0.01, Model 2), the coefficient of determination *R*^2^ for explaining the percentage of influence of the dependent variable in the fitted model was increased from 5 to 11%, and the *F* value was significant (*F* = 5.33, *p* < 0.001). This means that the fit of the model is good, that is, gamification will positively affect study engagement. Therefore, Hypotheses 1–4 are supported.

### Mediating effect analysis

When conducting the mediating effect test, this paper uses the test method of Baron and Kenny (1986). As shown in Table 3, the measurement results of Model 3 indicate that there is a significant positive effect between enjoyment and engagement (*β* = 0.37, *p* < 0.001). Model 4 adds a mediator, enjoyment, to Model 2. The results show that there was a significant mediating effect of enjoyment between gamification and study engagement (*ΔR*^2^ = 0.17, *F* = 8.82, *p* < 0.001). The regression coefficient of gamification on study engagement decreased (from 0.24 to 0.13, *p* < 0.01), and the significance also changed. After adding enjoyment, the relationship between gamification and study engagement is no longer significant and fully mediates the effect of gamification. Therefore, Hypothesis 5 is supported.

In addition, the mediating effect of self-efficacy was also verified. As shown in Table 3, the measurement results of Model 7 indicated that there was a significant positive effect between self-efficacy and study engagement (*β* = 0.40, *p* < 0.001). In Model 8, the mediating variable self-efficacy was added, and the results showed that self-efficacy had a significant mediating effect between gamification and engagement (*ΔR*^2^ = 0.19, *F* = 9.69, *p* < 0.001). The regression coefficient of gamification on engagement decreased (from 0.24 to 0.08, *p* < 0.01), and the significance also changed. After adding self-efficacy, the relationship between gamification and engagement is no longer significant; that is, self-efficacy played a complete mediating role in the effect of gamification on study engagement. Therefore, Hypothesis 6 is supported. To further validate our results, we conducted the bootstrap analyzes using the SPSS Process macro (Hayes, 2013). The results show that the 95% confidence intervals for the mediating effects of enjoyment and self-efficacy are (0.0159, 0.1048) and (0.0322, 0.1457), respectively. Therefore, Hypotheses 5 and 6 are further validated.

## Discussion

With the continuous development of discipline construction, the instructional design mode has become a hot issue in teaching reform. This paper integrates the concept of gamification design into teaching, which is currently a breakthrough in teaching. Gamification design teaching is of great significance in both theory and practice. Theoretical contributions and practice contributions can be listed as follows (Figure 1).

First, this paper provides a new perspective for classroom learning. Specifically, gamification design teaching uses a mix of different gamification elements, which improves the students’ initiative and enthusiasm to learn and focuses on the real needs of students in the learning process (Kim et al., 2020). Second, gamification design teaching can help teachers comprehensively understand and explore the interaction between various factors in the teaching process and their diversified forms of expression, which not only helps teachers grasp the essence and law of teaching but also helps students enhance the efficiency of learning (Prieto-Andreu et al., 2022). Third, gamification design fully reflects the active status of students in the learning process, which can emphasize the cultivation of students’ independent exploration spirit, form a good habit of learning and thus promote the optimization of the overall quality of students to a large extent.

Gamification design teaching is conducive to further promoting the quality of teaching (Hernández-Fernández et al., 2020; Arufe Giráldez et al., 2022). In detail, the way students receive the knowledge changes from passive learning to active learning. In addition, gamification design teaching helps solve problems of students in learning, stimulates students’ enthusiasm for learning and thus largely improves their academic performance. Second, students’ thinking ability and innovation ability can be enhanced through experiencing gamification design classes. Specifically, this teaching method improves students’ professional skills, knowledge and experience as well as professional quality. In addition, gamification design teaching, a successful motivational teaching, stimulates students’ challenging spirit and striving spirit to obtain a stronger sense of achievement. Last but not least, gamification design is suitable for the current situation of teaching. In detail, today’s society is extremely competitive and only have outstanding abilities can we stand out from the peer. Moreover, gamification design teaching is a teaching reform to make students master professional skills so that students have a specialty in learning to be precise and learning to use to some extent.

### Implications for teaching and learning

In gamified teaching, the educational concept advocated by teachers can be intuitively practiced so that teachers can fully pay attention to students’ behavior, clarify the central position of students in gamified teaching practice, and abandon teacher-centered gamification on this basis. The teaching concept lays the foundation for teachers to plan, design, and implement games according to their learning conditions. Second, teachers can mobilize students’ initiative through gamification teaching and constantly enrich the practice mode of gamification teaching, aiming to ensure that students can observe carefully, practice, express boldly, and think independently and make teaching gamification teaching methods more diverse. Finally, the continuous implementation of gamification teaching can make up for past shortcomings so that gamification teaching activities can keep pace with the times. In addition, design, planning, and teaching research are the abilities teachers must possess to complete the task of gamification teaching practice. Therefore, gamification teaching can lead teachers to design game goals, processes, evaluation standards, etc., according to students’ abilities, knowledge base, interests, life experience, growth environment, and other factors to lay the foundation for the smooth development of game activities.

The core function of gamification education is to change education from boring to exciting and vivid. Every teacher wants to achieve an active classroom atmosphere in which students are highly involved. However, doing both of these is not an easy task. Therefore, teachers need to understand the characteristics of different types of students to make targeted adjustments. Moreover, while the teacher pays attention to teaching, it increases the interest of the classroom and can transfer the attention of students who are easily distracted to the school. For example, in English and Chinese classes, teachers can choose a reading article, give each character a voice, and let students read out the content of the dialog with emotion. Alternatively, in the business management class, the teacher divides the students into several groups and assigns the students in the group to serve as CEO, financial director, human resources director, etc., only to allow the group members to operate the group of enterprises successfully. Through the above teaching experience, this paper found that teaching in the classroom is not only a pure knowledge explanation but that teachers can also bring various types of games into the classroom. Of course, which game is more suitable for your students depends on factors such as the number of students, the time of the game, and the degree of mastery of the students. In the education system, the most critical job for students is to learn knowledge, and teachers want to teach them as much knowledge as possible. However, for students to master knowledge better, faster, and more solidly, it is necessary to explore educational concepts that keep pace with the times.

In conclusion, using gamified design in teaching has made important contributions to theory and practice. The application of gamified design teaching can make learning more interesting, make students more active in learning and make teachers more initiative in teaching. In addition, gamified design teaching also has certain guiding significance to the teaching of various subjects, which is one of the innovations in teaching mode in order to achieve the dual effect of education (Geng et al., 2021).

### Limitations and future research directions

There are several limitations in this research. First, this research is cross-sectional in nature. Therefore, it provides little support for the causal inference. We suggest that future research can adopt the longitudinal design or experimental studies to validate the proposed relationships. Second, we just collected data from one university in China, which may limit the external validity of our findings. Future researchers can examine our framework in other context to test how gamification will enhance students’ study engagement. Finally, future research can include other cognitive or emotional states in the model to examine how students’ attitudes will be influenced in the gamified courses.

## Data availability statement

The raw data supporting the conclusions of this article will be made available by the authors, without undue reservation.

## Ethics statement

Ethical review and approval was not required for the study on human participants in accordance with the local legislation and institutional requirements. Written informed consent for participation was not required for this study in accordance with the national legislation and the institutional requirements.

## Author contributions

JC: developed the theoretical framework and worked on the literature review and manuscript writing. ML: developed the theoretical framework and worked on data collection and analysis. All authors contributed to the article and approved the submitted version.

## Conflict of interest

The authors declare that the research was conducted in the absence of any commercial or financial relationships that could be construed as a potential conflict of interest.

## Publisher’s note

All claims expressed in this article are solely those of the authors and do not necessarily represent those of their affiliated organizations, or those of the publisher, the editors and the reviewers. Any product that may be evaluated in this article, or claim that may be made by its manufacturer, is not guaranteed or endorsed by the publisher.
